# A Repertory of Convulsions

**Published:** 1890-12

**Authors:** 


A Repertory of Convulsions. E. M. Santee, M. D. New York: H. Hitchcock, M. D., 1890.

This handy little monograph, a reprint from the *Journal of Homœopathics*, should be in the hands of every Hahnenamannian, as it will save much time in studying convulsive affections. We need more of such, and in just such form, that they may always be with us.

